# Reducing Sedentary Time After Knee Replacement Using a Multicomponent mHealth Intervention: Randomized Controlled Trial

**DOI:** 10.2196/83148

**Published:** 2026-05-26

**Authors:** Christine A Pellegrini, Clare L Kennerley, Sara Wilcox, Jungwha Lee, Katherine DeVivo, Kailyn Horn, Scott Jamieson, Jeffrey Hopkins, Harley T Davis, J Benjamin Jackson III

**Affiliations:** 1 Department of Exercise Science Arnold School of Public Health University of South Carolina Columbia, SC United States; 2 Prevention Research Center University of South Carolina Columbia, SC United States; 3 Department of Preventive Medicine Northwestern University Chicago, IL United States; 4 School of Health Sciences Columbia College Columbia, SC United States; 5 Prisma Health Columbia, SC United States; 6 School of Medicine Columbia University of South Carolina Columbia, SC United States

**Keywords:** arthroplasty, sedentary behavior, mobile health, mHealth, physical activity

## Abstract

**Background:**

Total knee replacement (TKR) is a common surgery for end-stage knee osteoarthritis. Although reductions in pain and improvements in mobility occur after surgery, physical activity levels often do not change. Given the challenges of increasing physical activity in this population, targeting reductions in sedentary behavior may be a first step; however, no prior studies have examined the feasibility and effects of a sedentary reduction intervention after TKR.

**Objective:**

This study examined the effects of a 2-month multicomponent mobile health sedentary reduction intervention (*NEAT!2*) on sedentary time in adults with TKR.

**Methods:**

Adults (N=83; mean age 65.3, SD 9.4 years; mean BMI 32.7, SD 6.9 kg/m^2^; 62/83, 74.7% female; 64/83, 77.1% White) with a TKR ≤1 year ago were randomized to the *NEAT!2* group (n=42, 50.6%) or the attention-matched control group (n=41, 49.4%). The *NEAT!2* intervention focused on reducing sedentary time via a smartphone app designed to interrupt prolonged bouts (≥30 minutes) of sedentary behavior and through 5 coaching calls emphasizing goal setting and problem solving. The control group focused on surgery recovery via an app or website and 5 educational calls. Sedentary time, total physical activity, physical function, and pain were measured at 2 and 5 months. Linear mixed-effects models examined intervention effects and differences between groups at each time point.

**Results:**

Retention was 96% and 95% at 2 and 5 months, respectively, with no differences between groups. Participants in the *NEAT!2* group completed an average of 4.95 (SD 0.2) calls, used the app on an average of 40.3 (SD 13.8) days (out of 56 days), and received an average of 9.6 (SD 6.0) notifications per day. At 5 months, there was a significant increase in sit-to-stand transitions in the *NEAT!2* group and a substantial decrease in the control group, resulting in a significant difference between groups (mean difference 4.06, 95% CI 0.13-7.99; *P*=.04); however, the *NEAT!2* intervention did not result in significant effects on any of the other study outcomes at 2 or 5 months. Additionally, more days of app use were associated with greater increases in moderate-to-vigorous intensity physical activity (*r*=0.335; 95% CI 0.017-0.585; *P*=.04).

**Conclusions:**

This study highlights the challenges of reducing sitting time in adults with TKR. Future studies should explore alternative behavior change techniques across different levels of influence (eg, environmental and social) to support interventions implemented within the first year after TKR.

**Trial Registration:**

ClinicalTrials.gov NCT04482400; https://clinicaltrials.gov/ct2/show/NCT04482400

## Introduction

Total knee replacement (TKR) is one of the most common surgical procedures performed in the United States, and its prevalence is expected to continue rising rapidly [1,2]. TKR is considered an effective treatment for severe knee osteoarthritis, with most patients experiencing reduced pain and improved physical function and quality of life following the procedure [3-5]. However, postoperatively, most adults struggle to increase physical activity [6], further increasing their risk of all-cause mortality, cardiovascular disease, and disability, with less than 5% of adults with TKR meeting physical activity guidelines [7]. Several interventions have been developed to target physical activity after TKR; however, most have had limited or modest success in increasing steps or moderate-intensity physical activity [8-10].

In addition to struggling to increase physical activity, adults with TKR continue to spend excessive time in sedentary behaviors [11,12]. Sedentary behavior is defined as any waking behavior in a seated or reclining position at ≤1.5 metabolic equivalents of task [13]. Among inactive adults, higher sedentary time is associated with an increased risk of all-cause mortality [14]. Given the low levels of physical activity after surgery, targeting reductions in sedentary behavior may be a more feasible first-step approach to increasing overall activity in this population. Sedentary reduction interventions tailored to other populations, such as office workers, have been shown to be effective [15,16], and a recent study demonstrated that multicomponent sedentary behavior interventions were well accepted and effective in older adults [17]; however, no prior studies have examined the effects of a sedentary reduction intervention after TKR.

The purpose of this study was to examine the effects of a multicomponent mobile health (mHealth) sedentary reduction intervention on sedentary time in adults with TKR at the end of the intervention (2 months) and following a 3-month maintenance period (5 months). Changes in physical activity, physical function, and pain were also examined at each time point. Additionally, this study examined the relationship between adherence to the mHealth program and changes in sedentary time, physical activity, physical function, and pain.

## Methods

### Study Design

This study was a randomized clinical trial, and full study details have been previously published [18]. Participants with TKR were randomized to either the mHealth sedentary reduction intervention (the *NEAT!2* group) or an attention control group. Outcomes were assessed at baseline, at the end of the intervention (2 months), and following a 3-month maintenance period.

### Participants

Participants were recruited and enrolled between August 2020 and April 2024. Initially, participants were recruited at least 7 days prior to knee replacement and then started the program 4 weeks after surgery; however, due to recruitment challenges because of the COVID-19 pandemic, recruitment and eligibility criteria were modified in October 2021. Following the change, participants were eligible if they had a unilateral TKR within <1 year of the baseline assessment. Additional inclusion criteria included being aged 40 to 79 years, having a smartphone that was accessible and near them most of the day, being willing to download the study smartphone apps, self-reporting spending at least 7 hours a day sitting, and being English-speaking. Participants were excluded if they had any contraindications to activity, had a mobility-limiting comorbidity (eg, spinal stenosis), were scheduled for surgery (eg, TKR on the contralateral knee) within the next 6 months, or did not have ≥4 days of valid ActiGraph (ARCHIMED) accelerometer wear (≥10 hours/day) at baseline. There was no racial or gender bias in the selection of participants.

Participants were primarily recruited from orthopedic centers within a local health care system in Columbia, South Carolina. Study candidates were contacted via postcards, mailings, emails, or telephone calls. Additional recruitment strategies included flyers posted on the university campus or in local businesses or clinics, emails to university faculty and staff, and social media. All recruitment materials directed interested candidates to complete an online or telephone screening with study staff. Eligible candidates were invited to an in-person session to review complete study details and complete the written informed consent process if interested and eligible.

### Randomization

After completing the baseline assessment and verifying accelerometer wear time, participants were contacted via telephone to remind them of the study details. Additionally, to promote retention and prevent differential attrition, the benefits and barriers to both general research study participation and specific study participation were reviewed, and participants were asked to discuss their personal perspectives on the pros and cons of being randomized to either study condition [19]. Participants who expressed continued interest in participating were randomized to one of two conditions: (1) *NEAT!2* or (2) control. Randomization was created by the biostatistician, and participants were stratified by age (<65 years and ≥65 years). Participants were randomly assigned in a 1:1 ratio to either the *NEAT!2* group or the control group using permuted block sizes of 2 or 4.

### Randomized Conditions

#### NEAT!2

**Figure 1:**
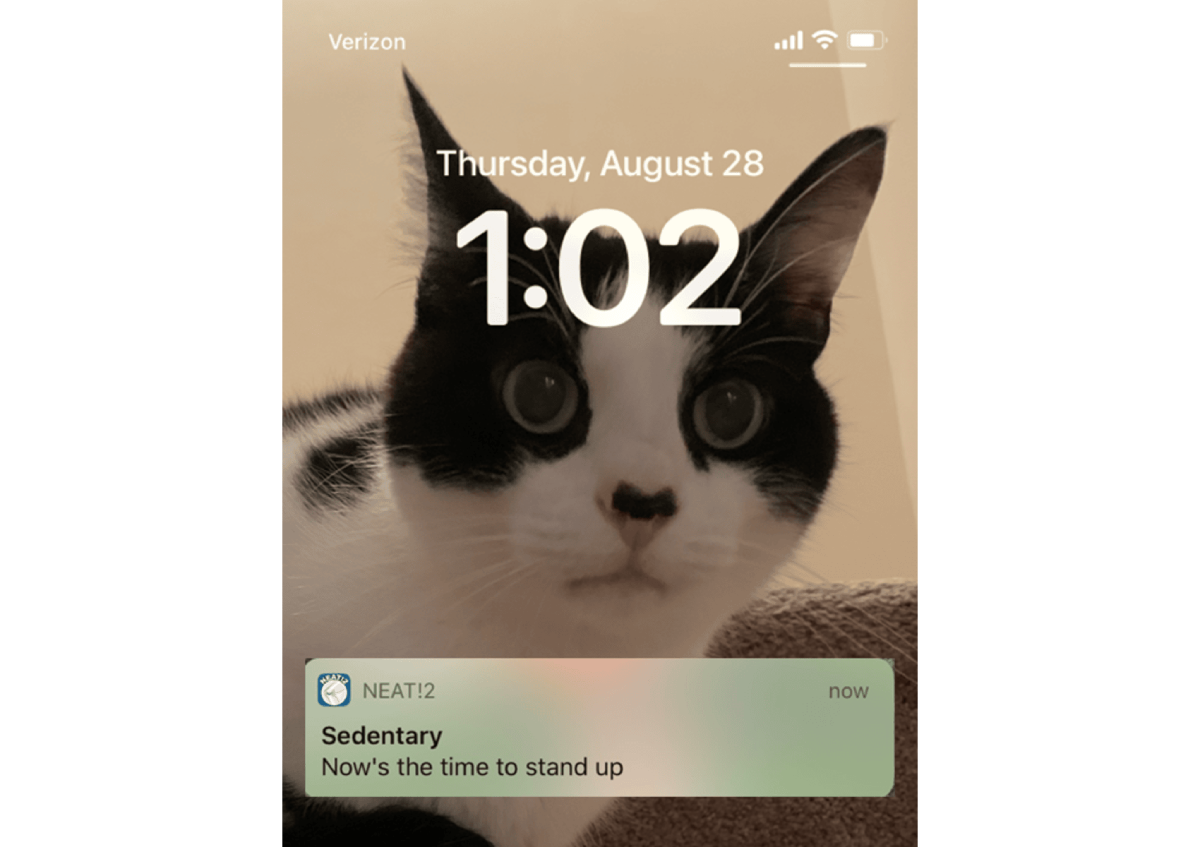
Screenshot of an example NEAT!2 app notification.

Participants randomized to *NEAT!2* received the mHealth and coaching intervention guided by the Dual-Process Theory [20,21]. Participants were given goals to reduce sedentary time, starting with a reduction of 30 minutes per day and progressing every 2 weeks to a final goal of reducing sedentary time by 90 minutes per day. To help participants reach this goal, the *NEAT!2* app was turned on after randomization and displayed the overall daily goal. The app was designed to detect prolonged sedentary time using the accelerometer and activity recognition libraries on the participant’s Android or iOS device. Specifically, when 30 minutes of continuous inactivity was detected, the app generated a sound or vibration and placed a notification on the participant’s phone (Figure 1). The notifications varied in content, but each instructed participants to take a break from sitting, either by standing or walking for at least 2 minutes. The *NEAT!*2 prompts were designed to target automatic (implicit) processes.

In conjunction with the *NEAT!2* app, controlled (conscious) processes were targeted through goal setting, education, problem solving, and behavioral feedback. Participants received a coaching call every 2 weeks during the 2-month intervention (5 calls in total). Calls were intended to last 10 to 15 minutes and focused on app use, goal setting, and problem solving around reducing sedentary time, with coaches guided by semistructured call scripts. All coaches had a background in exercise science, psychology, public health, or a related field.

Between the 2- and 5-month assessments, participants received monthly phone calls from research staff members who did not conduct the coaching calls. These calls focused solely on surgery recovery and were designed to help with retention during the follow-up period. Participants were given the option to continue using the *NEAT!2* app during this period; however, use of the app or sedentary time was not discussed on calls.

#### Attention-Matched Control

Participants randomized to the control group received an attention-matched education program focused on surgery recovery, which included a smartphone app or website (My Knee Guide) and regular calls. Control participants were encouraged to use the app or website to plan recovery activities and read about others’ experiences after TKR. Participants also received calls from the study coaches every 2 weeks (5 calls in total) to discuss surgery recovery and education after TKR (eg, dealing with fatigue, emotional self-care, and reducing injuries at home). If participants brought up unintended content (eg, sedentary behavior or physical activity), coaches were trained to redirect the conversation.

Similar to *NEAT!2* participants, control participants received monthly phone calls on surgery recovery between the 2- and 5-month assessments. Following the 5-month assessment, the control group was offered access to the *NEAT!2 app.*

### Treatment Fidelity

For both groups*,* all coaching sessions were audiotaped, and a 15% sample of calls was rated every quarter. Fidelity assessments evaluated unintended and intended content based on randomized conditions. If fidelity fell below 80% or coaches discussed unintended content, coaches were retrained.

### Measures

Assessments were completed either in an orthopedic clinic or a campus research center at baseline and at 2-month and 5-month follow-ups. Study assessors were blinded to group allocation and had undergone training on all assessment procedures. All questionnaires were completed online via REDCap (Research Electronic Data Capture) [22].

### Outcomes

The primary outcome of the study was the percentage of time spent in sedentary behavior as assessed by ActiGraph at 2 months. Secondary outcomes included total physical activity time (light-, moderate-, and vigorous-intensity physical activity), physical function, and pain. Exploratory outcomes included sedentary time assessed by activPAL, knee symptoms, general health, sleep disturbance, and habit strength.

### Sedentary Time and Physical Activity

At each assessment, participants were asked to wear an ActiGraph GT9X Link waist-worn accelerometer for 7 days during waking hours (except during water-based activities). A minimum of 4 days of valid wear time consisting of at least 10 hours/day was required for inclusion in analyses [23]. Nonwear time was defined as ≥90 minutes with 0 activity counts, allowing for up to 2 minutes of <100 counts/minute [24]. Sedentary time was defined as <100 counts/minute, and total activity as ≥100 counts/minute [23]. Average daily sedentary time (minutes/day), percentage of the waking day spent in sedentary time, and weekly total physical activity (light-, moderate-, and vigorous-intensity activity) were calculated within the ActiLife (version 6; Ametris) software.

In addition to ActiGraph monitors, the activPAL software (version 8; PAL Technologies) and Sedentary Time and Activity Reporting Questionnaire [25] were used as exploratory measures of sedentary time at each time point. The activPAL monitor was waterproofed and taped onto participants’ thighs using waterproof tape. Participants were asked to wear the monitor for 24 hours a day during each 7-day period. A minimum of 4 days of valid wear time, defined as 24 hours, was required for inclusion in analyses. The time spent sitting or lying, standing, and walking; transitions; and step counts were determined using the activPAL. The Sedentary Time and Activity Reporting Questionnaire [25] was also administered and is a self-reported measure of habitual sedentary behavior. The average number of hours per day spent sitting on weekdays and weekends was calculated.

### Physical Function

Physical function was assessed using the Chair Stand Test, Timed Up and Go test, and 6-minute walk test. All physical function tests were completed following the Osteoarthritis Research Society International–recommended procedures [26]. During the Chair Stand Test, participants were asked to complete as many chair stand repetitions as possible during a 30-second period. The Timed Up and Go test assessed the time, in seconds, taken to rise from a chair, walk 3 meters, turn, walk back to the chair, and sit down. The 6-minute walk test evaluated the maximal distance, in feet, that a participant could walk during a 6-minute period.

### Patient-Reported Outcomes

Pain and related self-reported outcomes were assessed using the Western Ontario and McMaster Universities Osteoarthritis Index [27] and Knee Injury and Osteoarthritis Outcome Score [28]. The Western Ontario and McMaster Universities Osteoarthritis Index is a 15-item survey assessing pain, stiffness, and functional limitations over the last 48 hours on a 5-point Likert scale. Scores range from 0 to 20 for pain, 0 to 8 for stiffness, and 0 to 68 for physical function, with higher scores indicating more symptoms. The Knee Injury and Osteoarthritis Outcome Score assesses overall knee health and includes 5 subscales: pain, symptoms, activities of daily living, sport and recreation, and knee-related quality of life. Scores on each subscale range from 0 (no knee issues) to 100 (extreme knee issues). General health, sleep disturbance, and mobility were also assessed using Patient-Reported Outcomes Measurement Information System computer-adaptive tests via REDCap. Each survey generates a *t* score, where scores <50 indicate poorer outcomes, while scores >50 reflect better outcomes.

Habit strength was assessed with 4 items for sitting, stretching, and exercising from the Self-Report Habit Index [29]. Participants were asked to rate each behavior from 1 (strongly disagree) to 7 (strongly agree) on the following items: “I do automatically,” “I do without having to consciously remember,” “I do without thinking,” and “I start doing before I realize I’m doing it.” Higher scores indicate greater habit strength.

### Demographics and Intervention Adherence

Demographics and medical history were obtained at baseline. Height (m) and weight (kg) were measured in light clothing and without shoes at each time point. BMI was subsequently calculated as kg/m^2^.

Adherence to the *NEAT!2* intervention was assessed by the percentage of coaching calls completed (completed calls/5 total calls), the total days the *NEAT!2* app was used (56 possible days), and the response to *NEAT!2* notifications. Total days the *NEAT!2* app was used were determined as the number of unique days participants received notifications through the app. The response to *NEAT!2* notifications was determined by the percentage of notifications for which movement or activity was detected by the app within 5 minutes of notification delivery.

### Adverse Events

Injuries and illnesses were assessed at each assessment, as well as during follow-up calls. All adverse events were monitored and rated based on their severity, expectedness, and relatedness to the study.

### Statistical Analysis

Descriptive statistics were used to describe participants’ baseline characteristics. Mean (SD) for continuous variables and frequency (percentage) for categorical variables were reported. Linear mixed-effects models were used to evaluate whether the *NEAT!2* sedentary reduction intervention led to greater improvements in sedentary time (ActiGraph minutes/day and percentage of the day spent sedentary), physical activity, physical function, and pain than the control group at 2 and 5 months. Models included group (*NEAT!2* vs control), time (baseline, 2-month follow-up, and 5-month follow-up), and group×time interaction terms. Models accounted for repeated measures with a random intercept to allow for baseline differences. Estimated regression coefficients and corresponding 95% CIs were computed for each outcome. Potential confounders included age, sex, BMI, and comorbidities. ActiGraph total and moderate-to-vigorous intensity physical activity were adjusted for monitor wear time. To examine the association between adherence to the *NEAT!2* intervention and changes in outcomes (sedentary time, physical activity, physical function, and pain), bootstrap Spearman correlation coefficients and corresponding 95% Cis, accounting for the small sample size, were computed between adherence to the *NEAT!2* intervention and changes in outcomes. All hypothesis tests were 2-sided with statistical significance set at *P*<.05. No adjustments were made for multiple comparisons. All statistical analyses were performed in SAS (version 9.4; SAS Institute Inc).

The sample size calculations have been reported previously [18]. In brief, having 40 participants per group complete the 2-month and 5-month assessments would provide 90% power to detect a difference of 8.1% reduction in sedentary time (ie, effect size of 0.7) between participants in the *NEAT!2* and control groups. A reduction of 8% was chosen as it is approximately equivalent to a 90-minute reduction in sedentary time over the course of a waking day.

### Ethical Considerations

All study procedures were approved by the University of South Carolina’s Institutional Review Board (approval number Pro00092132) on September 19, 2019, and all procedures were conducted in accordance with the Helsinki Declaration of 1975. All participants provided written informed consent before participation. The trial was registered on Clinicaltrials.gov (NCT04482400). A CONSORT checklist is provided in Multimedia Appendix 1. Participants received US $15 for completing each follow-up assessment, and all research data were deidentified.

## Results

### Participant Characteristics, Retention, and Safety

**Figure 2:**
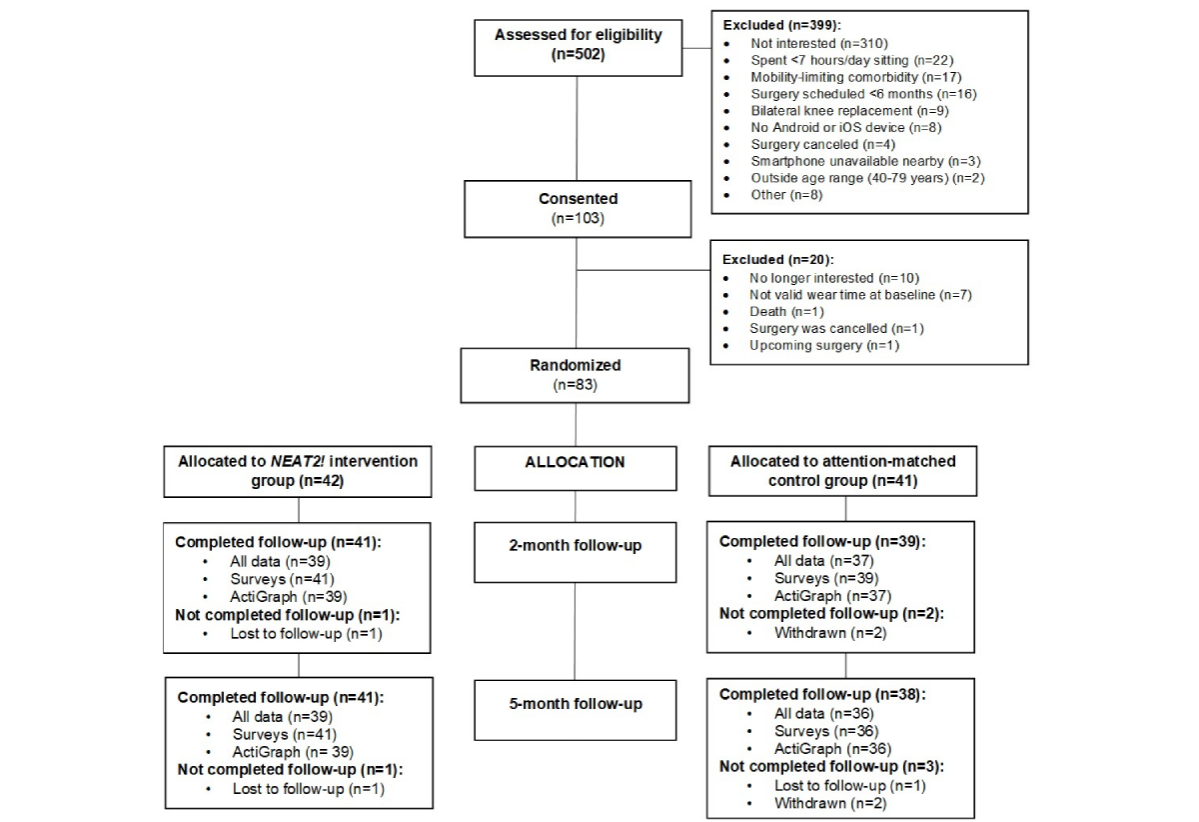
CONSORT (Consolidated Standards of Reporting Trials) flow diagram for the study.

Of the 502 individuals screened for eligibility, 103 (20.5%) were eligible and provided consent (Figure 2). The primary reasons participants were excluded were lack of interest (n=310, 77.7%), reporting sitting <7 hours/day (n=22, 5.5%), or having a mobility-limiting comorbidity (n=17, 4.3%). Following consent, 83 (80.6%) participants were eligible and underwent randomization (n=42, 50.6% to the *NEAT!2* group and n=41, 49.4% to the control group). Retention was 96% and 95% at 2-month follow-up and 5-month follow-up, respectively, with no differences between groups at either time point.

Participants in the *NEAT!2* and control groups had similar characteristics at baseline (Table 1). Participants had a mean age of 65.3 (SD 9.4) years, were primarily female (n=62, 74.7%) and White (n=64, 77.1%), and had an average BMI of 32.7 (SD 6.9) kg/m^2^. Most participants (n=44, 53%) were employed full-time or part-time. The most common medical conditions included hypertension (n=48, 57.8%), depression or anxiety (n=27, 32.5%), sleep apnea (n=22, 26.5%), and diabetes (n=18, 21.7%). In total, 9 (10.8%) participants were recruited prior to surgery (mean 12.0, SD 3.5 days before surgery), and 74 (83.2%) participants were recruited a median of 213 (IQR 87-300.5, range 38- 351) days after surgery. During the study period, 32 adverse events (2 prior to randomization, 8 in the control group, and 22 in the *NEAT!2* group) and 6 serious adverse events (3 in the control group and 1 in the *NEAT!2* group) occurred*.* The serious adverse events included 1 death and 5 surgeries or hospitalizations. Only 1 adverse event (nerve entanglement in the back) was deemed possibly related to the intervention; all others were deemed unrelated to the study.

**Table 1:** Baseline characteristics of the participants by full sample and by group allocation.

Characteristics			Total sample (n=83)		*NEAT!2* group (n=42)		Control group (n=41)
Age (years), mean (SD)			65.3 (9.4)		65.5 (9.8)		65.1 (9.0)
**Sex, n (%)**							
	Female	62 (74.7)		32 (76.2)		30 (73.2)	
	Male	21 (25.3)		10 (23.8)		11 (26.8)	
BMI (kg/m^2^), mean (SD)			32.7 (6.9)		33.7 (7.1)		31.7 (6.6)
Days since knee surgery, mean (SD)			175.3 (121.8)		192.8 (122.6)		157.4 (119.8)
**Race, n (%)**							
	Black or African American	19 (22.9)		11 (26.2)		8 (19.5)	
	White	64 (77.1)		31 (73.8)		33 (80.5)	
Ethnicity: not Hispanic or Latinx, n (%)			83 (100)		42 (100)		41 (100)
**Marital status, n (%)**							
	Married	51 (61.4)		30 (71.4)		21 (51.2)	
	Not married	32 (38.6)		12 (28.6)		20 (48.8)	
**Education, n (%)**							
	Below a college degree	26 (31.3)		11 (26.2)		15 (36.6)	
	College degree or above	57 (68.7)		31 (73.8)		26 (63.4)	
**Employment status, n (%)**							
	Full-time employment	33 (39.8)		17 (40.5)		16 (39.0)	
	Part-time employment	11 (13.2)		5 (11.9)		6 (14.6)	
	Retired or not working	39 (47.0)		20 (47.6)		19 (46.3)	
**Household income (US $), n (%)**							
	<100,000	41 (49.4)		22 (52.4)		19 (46.3)	
	≥100,000	33 (39.8)		16 (38.1)		17 (41.5)	
	Prefer not to answer	9 (10.8)		4 (9.5)		5 (12.2)	
**Comorbidities, n (%)**							
	Diabetes	18 (21.7)		9 (21.4)		9 (22.0)	
	Cancer	12 (14.4)		8 (19.0)		4 (9.8)	
	Depression and/or anxiety	27 (32.5)		15 (35.7)		12 (29.3)	
	High blood pressure	48 (57.8)		24 (57.1)		24 (58.5)	
	Thyroid problems	17 (20.5)		10 (23.8)		7 (17.1)	
	Sleep apnea	22 (26.5)		12 (28.6)		10 (24.4)	

### Intervention Effects on Primary and Secondary Outcomes

Valid ActiGraph data were provided by 82 (98.8%) participants at baseline (mean 6.6, SD 0.8 valid days), 76 (91.6%) participants at 2 months (mean 6.4, SD 0.9 valid days), and 75 (90.4%) participants at 5 months (mean 6.4, SD 0.9 valid days). Valid activPAL data were provided by 81 (97.6%) participants at baseline (mean 6.5, SD 0.7 valid days), 74 (89.2%) participants at 2 months (mean 6.2, SD 0.6 valid days), and 74 (89.2%) participants at 5 months (mean 6.2, SD 0.8 valid days). The intervention effects on sedentary behavior and physical activity outcomes are shown in Table 2. At baseline, the average percentage of the waking day spent in sedentary time was 73.26% (SD 7.47%) in the *NEAT!2* group and 74.13% (SD 6.17%) in the control group. At 5 months, ActiGraph-assessed moderate-to-vigorous physical activity (MVPA; mean difference −24.23, 95% CI −46.91 to −1.55; adjusted for weekly wear time; *P=*.04) and activPAL sit-to-stand transitions (mean difference 4.06, 95% CI 0.13-7.99; *P=*.04) were different between groups. The *NEAT!2* intervention did not result in statistically significant effects on any other sedentary behavior or physical activity outcomes at 2 or 5 months, and no other differences were observed between groups. The intervention effects on physical function and patient-reported outcomes are shown in Table 3. Exploratory outcomes are shown in Multimedia Appendix 2. There were also no significant effects across time or by group.

**Table 2:** Sedentary behavior and physical activity outcomes at baseline, at 2-month follow-up, and at 5-month follow-up by group allocation.

Outcomes and time points				*NEAT!2* group (n=42), mean (SE)		Control group (n=41), mean (SE)		Group×time interaction effects in mixed-effects models		
								β coefficient (95% CI)		*P* value
**ActiGraph**										
	**Percentage of time sedentary (%** **per day** **)**									
		Baseline	73.26 (1.06)		74.05 (1.07)		—^a^		—	
		2-month follow-up	73.55 (1.08)		74.36 (1.09)		−0.02 (−2.36 to 2.33)		.99	
		5-month follow-up	74.73 (1.08)		73.74 (1.10)		1.78 (−0.57 to 4.14)		.14	
	**Sedentary time (minutes per day^b^)**									
		Baseline	658.77 (12.30)		659.41 (12.06)		—		—	
		2-month follow-up	644.18 (12.61)		658.90 (12.37)		−0.76 (−21.28 to 19.75)		.94	
		5-month follow-up	642.90 (12.71)		673.56 (12.37)		15.39 (−5.23 to 36.01)		.14	
	**Total physical activity (light-, moderate-, or vigorous-intensity; minutes per week^b^)**									
		Baseline	1642.76 (72.66)		1471.93 (73.93)		—		—	
		2-month follow-up	1530.88 (74.07)		1390.24 (75.37)		−14.86 (−141.59 to 111.86)		.82	
		5-month follow-up	1510.26 (74.07)		1424.61 (75.83)		−96.25 (−223.63 to 31.12)		.14	
	**Moderate-to-vigorous physical activity (minutes per week^b^)**									
		Baseline	78.67 (86.06)		51.08 (71.06)		—		—	
		2-month follow-up	58.51 (74.62)		49.76 (60.68)		−18.51 (−41.07 to 4.06)		.11	
		5-month follow-up	60.67 (89.10)		57.81 (65.74)		−24.23 (−46.91 to −1.55)		.04	
	**Wear time (minutes per day)**									
		Baseline	900.42 (64.65)		888.42 (81.99)		—		—	
		2-month follow-up	972.74 (193.88)		923.29 (209.03)		18.52 (−12.32 to 49.36)		.88	
		5-month follow-up	944.02 (120.31)		924.37 (228.56)		18.62 (−12.37 to 49.61)		.73	
**activPAL**										
	**Sitting time (minutes per day)**									
		Baseline	626.20 (17.95)		633.12 (18.17)		—		—	
		2-month follow-up	630.46 (18.22)		611.82 (18.79)		25.55 (−16.71 to 67.81)		.23	
		5-month follow-up	623.79 (18.45)		622.74 (18.54)		7.96 (−34.25 to 50.18)		.71	
	**Standing time (minutes per day)**									
		Baseline	213.13 (11.82)		191.05 (11.96)		—		—	
		2-month follow-up	219.87 (11.96)		196.72 (12.29)		1.08 (−23.41 to 25.57)		.93	
		5-month follow-up	221.51 (12.08)		207.17 (12.16)		−7.73 (−32.19 to 16.74)		.53	
	**Walking time (minutes per day)**									
		Baseline	79.52 (4.52)		73.34 (4.58)		—		—	
		2-month follow-up	78.81 (4.57)		75.01 (4.68)		−2.38 (−10.99 to 6.23)		.58	
		5-month follow-up	78.41 (4.61)		76.18 (4.64)		−3.95 (−12.55 to 4.66)		.37	
	**Sit-to-stand transitions (transitions per day)**									
		Baseline	45.82 (1.86)		47.21 (1.89)		—		—	
		2-month follow-up	48.38 (1.89)		46.46 (1.94)		3.31 (−0.62 to 7.24)		.10	
		5-month follow-up	47.66 (1.91)		45.00 (1.92)		4.06 (0.13 to 7.99)		.04	
	**Steps (steps per day)**									
		Baseline	5959.90 (368.33)		5325.10 (372.83)		—		—	
		2-month follow-up	5920.49 (372.12)		5413.79 (381.33)		−128.10 (−823.13 to 566.93)		.72	
		5-month follow-up	5956.20 (375.19)		5615.94 (377.97)		−294.54 (−988.81 to 399.72)		.40	

^a^Not applicable.

^b^Adjusted for either daily or weekly wear time.

**Table 3:** Physical function and patient-reported outcomes at baseline, at 2-month follow-up, and at 5-month follow-up by group allocation.

Outcomes and time points			*NEAT!2* group (n=42), mean (SE)		Control group (n=41), mean (SE)		Group×time interaction effects in mixed-effects models		
							β coefficient (95% CI)		*P* value
**6-minute walk test (feet)**									
	Baseline	1231.36 (46.69)		1207.42 (47.25)		—^a^		—	
	2-month follow-up	1328.44 (46.84)		1330.05 (47.85)		−7.79 (−29.70 to 14.12)		.48	
	5-month follow-up	1342.40 (46.84)		1342.89 (48.15)		−7.45 (−29.61 to 14.70)		.51	
**30-second Chair Stand Test (repetitions)**									
	Baseline	9.48 (0.43)		9.34 (0.43)		—		—	
	2-month follow-up	10.85 (0.43)		10.39 (0.44)		0.33 (−0.46 to 1.12)		.41	
	5-month follow-up	10.54 (0.43)		10.40 (0.44)		0.00 (−0.79 to 0.80)		.996	
**Timed Up and Go test (seconds)**									
	Baseline	10.28 (0.37)		10.20 (0.38)		—		—	
	2-month follow-up	9.54 (0.38)		9.45 (0.38)		0.01 (−0.74 to 0.77)		.97	
	5-month follow-up	9.25 (0.38)		9.54 (0.39)		−0.37 (−1.13 to 0.39)		.34	
**WOMAC^b^** **pain score**									
	Baseline	5.21 (0.53)		5.37 (0.54)		—		—	
	2-month follow-up	2.98 (0.53)		3.67 (0.54)		−0.54 (−1.63 to 0.55)		.33	
	5-month follow-up	3.10 (0.53)		3.03 (0.55)		0.23 (−0.87 to 1.32)		.69	
**WOMAC stiffness score**									
	Baseline	3.07 (0.27)		3.15 (0.27)		—		—	
	2-month follow-up	2.55 (0.27)		2.17 (0.28)		0.45 (−0.23 to 1.14)		.19	
	5-month follow-up	2.28 (0.27)		1.95 (0.28)		0.41 (−0.28 to 1.10)		.24	
**WOMAC function score**									
	Baseline	17.95 (1.64)		16.68 (1.66)		—		—	
	2-month follow-up	11.96 (1.66)		12.45 (1.68)		−1.76 (−5.59 to 2.08)		.37	
	5-month follow-up	11.33 (1.66)		11.23 (1.70)		−1.18 (−5.05 to 2.70)		.55	

^a^Not applicable.

^b^WOMAC: Western Ontario and McMaster Universities Osteoarthritis Index (higher scores on WOMAC indicate more knee symptoms).

A sensitivity analysis was performed including covariates such as BMI, marital status, cancer, depression, and the interaction between baseline physical activity and BMI. Effect estimates were similar across all covariate specifications. Compared with the primary outcome of ActiGraph percentage of time sedentary (group × time interaction effect −0.02, 95% CI −2.36 to 2.33 at 2M; 1.78, 95% CI −0.57 to 4.14 at 5M), estimates from the model adjusted for BMI, marital status, cancer, and depression (0.01, 95% CI −2.34 to 2.35 at 2M; 1.82, 95% CI −0.54 to 4.17 at 5M) and the model accounting for interaction of baseline low physical activity and BMI (0.28, 95% CI −2.01 to 2.57 at 2M; 2.18, 95% CI −0.12 to 4.49 at 5M) did not change conclusions. After accounting for covariates, the *NEAT!2* intervention did not result in statistically significant improvement on any of the other sedentary behavior or physical activity outcomes at 2 or 5 months.

### Intervention Engagement

On average, participants completed an average of 4.8 (SD 0.6) calls of the possible 5 calls, with no difference between randomized groups. Average call times for *NEAT!2* participants (mean 13.0, SD 7.2 minutes) were greater than those for participants in the control group (mean 10.65, SD 4.8 minutes; *P*<.001). Participants in the *NEAT!2* condition used the app for an average of 40.3 (SD 3.82) days over the 56-day intervention, with participants receiving an average of 9.7 (SD 6.0) notifications per day. Participants responded to *NEAT!2* notifications (movement detected by the smartphone) on an average of 9.7% (SD 7.8%; range 2%-42%) of occasions, with an average time between notification and movement of 2.4 (0.4) minutes.

### Association Between Intervention Adherence and Changes in Outcomes

A higher percentage of days of *NEAT!2* app use was associated with greater increases in MVPA (*r*=0.335; 95% CI 0.017-0.585; *P*=.04). There were no other significant relationships between adherence to call completion or response to *NEAT!2* notifications and changes in sedentary time, physical activity, physical function, and pain.

## Discussion

This study examined the effects of a multicomponent mHealth sedentary reduction intervention (*NEAT!2*) on sedentary time in adults with recent TKR. Additionally, this study examined the dose-response relationship between adherence to the mHealth program and changes in each outcome. Despite high adherence to the program, the *NEAT!2* intervention only led to improvements in sit-to-stand transitions assessed by activPAL at 5 months. The intervention did not result in any other statistically significant changes in the study outcomes of sedentary time, physical activity, physical function, or pain. Results did not change even after adjusting for covariates. Although there were no between-group differences in outcomes, there was a positive association between adherence to the *NEAT!2* app and MVPA, suggesting that those who used the app more frequently had greater increases in MVPA.

Although some previous interventions have been effective in reducing sedentary time in non-TKR populations [15-17], the literature is mixed, particularly among older adults [30]. A recent systematic review by Chastin et al [30] highlighted the uncertainties regarding whether sedentary reduction interventions can effectively reduce sedentary time in older adults; however, the review only included 7 studies. The lack of changes in sedentary time in this *NEAT!2* mHealth intervention among adults <1 year after TKR is consistent with this review [30], highlighting the challenges of reducing sitting time in an older clinical population, as well as the need for more studies examining reductions in sedentary behavior in older adults.

This intervention was guided by the Dual-Process Theory [20,21] and targeted both automatic and conscious individual-level behaviors. The behavior change techniques implemented in this study included strategies, such as reminders to interrupt sedentary behavior (targeting automatic processes) and goal setting (targeting conscious processes), to target total sedentary time. Previously, mHealth interventions, including disruptive smartphone apps designed to interrupt sedentary behavior, have shown effectiveness in reducing total sedentary time among various populations, including individuals with overweight or obesity and diabetes [15,31]; however, these strategies may not have been sufficient to change daily sitting time in adults after TKR within this brief 2-month intervention. The intervention increased sit-to-stand transitions at 5 months, but to change total sedentary time, providing real-time feedback may be necessary, as this approach has been an effective behavior change technique for physical activity [32]. Unfortunately, there were no devices at the time, to our knowledge, that could accurately provide feedback on sitting time. Furthermore, many effective sedentary reduction programs have included environmental changes and the provision of standing desks, primarily for office workers [33]. Although this approach may work for working adults, approximately half of the participants in this study were retired or not working; thus, standing desks may not be an effective strategy for this population. Environmental strategies for retired adults could include modifying the home by adding standing-friendly spaces (eg, high tables or counters) for activities such as puzzles, rearranging furniture and living space to encourage standing and light movement, or incorporating visual cues or reminders (eg, notes near the television) to break up sitting time. Future interventions may need to increase the focus on environmental, as well as cultural and social factors that could be influencing sedentary behavior more than individual-level factors [30,34].

Potential postoperative and COVID-19 pandemic–related barriers faced by the current participants may have also reinforced sedentary behaviors [35] and prevented the intervention from being effective. Additionally, previous research has suggested that adults with TKR and osteoarthritis are unaware of the health benefits of light-intensity activity and often think that sedentary time should be replaced with moderate-intensity activity [34]. This intervention primarily promoted light-intensity activity such as standing or light walking; however, the results suggested that the more days the app was used, the greater the increase in MVPA. Despite coaches encouraging the replacement of sedentary time with light-intensity activity, participants may still have focused on replacing sedentary time with MVPA such as through brisk walk. Although increasing MVPA would provide substantial health benefits, it is unreasonable to think that all excess sedentary time could be replaced with MVPA. More effort is needed to raise awareness of the health consequences of excess sedentary time and the benefits of replacing sedentary time with activity of any intensity in older adults [36].

This study had several strengths. In addition to being one of the first studies to examine a sedentary reduction program after TKR, the use of multiple objective and subjective measurement tools provides further evidence of the challenges and inconsistencies in measuring sedentary time in this population. However, this study has also had several limitations. The COVID-19 pandemic resulted in necessary changes to recruitment whereby some participants completed baseline testing before surgery, and others completed testing postoperatively. Although this was accounted for in the analyses, the COVID-19 pandemic may have had other effects on participants and outcomes in both the intervention and control groups. Additionally, the prevalence of depression and/or anxiety (32.5%) was higher than anticipated and may have affected adherence to the intervention. The sample size was smaller than anticipated, resulting in lower power to detect changes and there was potential for inter- and intraindividual variation in capturing habitual sedentary and physical activity behaviors, which may have influenced the results. Furthermore, there is likely an error in the determination of responsiveness to the *NEAT!2* notifications, as the determination of response was based on movement detected on the smartphone. It is possible that participants may have stood up or walked around but did not move their phone; thus, the app would not have been able to detect that response. Additionally, the ActiGraph activity monitor cannot distinguish sitting from standing. Future studies may need to consider using more innovative wearables that can detect postural changes.

A multicomponent mHealth sedentary reduction intervention led to changes at 5M in sit-to-stand transitions but was not effective in reducing sedentary time in adults who had undergone TKR; however, participants who used the app more frequently throughout the intervention had greater increases in MVPA. This study highlights the challenges of reducing sitting time in this population and presents direction for future studies, which should explore alternative behavior change techniques across different levels of influence (eg, environmental and social) within interventions during the first year after TKR.

## Appendix

Multimedia Appendix 1 CONSORT (Consolidated Standards of Reporting Trials) checklist.

Multimedia Appendix 2 Exploratory outcomes by randomized condition at 2 and 5 months.

## Abbreviations

mHealth: mobile health
MVPA: moderate-to-vigorous physical activity
REDCap: Research Electronic Data Capture
TKR: total knee replacement

## Data Availability

The datasets generated or analyzed during this study are available from the corresponding author on reasonable request.
