# Supplementary material for: Reducing Sedentary Time After Knee Replacement Using a Multicomponent mHealth Intervention: Randomized Controlled Trial
Source: JMIR Mhealth Uhealth. 2026 May 26;14:e83148. doi: 10.2196/83148 (PMC13250498; doi:10.2196/83148)
Supplement: Multimedia Appendix 2 [file mhealth_v14i1e83148_app2.docx]

Appendix Table 1. Exploratory outcomes by randomized condition at 2 and 5 months

| **Outcome** | **Time** | ***NEAT!2* (n=42)** | **Control (n=35)** | **Group*Time Interaction Effects in Mixed Effects Models** | | |
| --- | --- | --- | --- | --- | --- | --- |
|  |  | **Mean (SE)** | **Mean (SE)** | **β** | **95% CI** | ***P*** |
| SIT-Q Sitting time on weekday, hours/day | BL | 11.66 (0.65) | 11.31 (0.66) | - |  |  |
|  | 2M | 10.27 (0.65) | 9.70 (0.66) | 0.22 | -1.79, 2.23 | 0.831 |
|  | 5M | 9.98 (0.65) | 10.15 (0.66) | -0.52 | -2.53, 1.49 | 0.610 |
| SIT-Q Sitting Time on weekend, hours/day | BL | 10.34 (0.66) | 10.42 (0.67) | - |  |  |
|  | 2M | 9.53 (0.66) | 9.19 (0.67) | 0.42 | -1.81, 2.65 | 0.709 |
|  | 5M | 9.58 (0.66) | 9.32 (0.67) | 0.34 | -1.89, 2.56 | 0.767 |
| KOOS Pain | BL | 69.51 (2.65) | 69.11 (2.68) | - |  |  |
|  | 2M | 80.59 (2.66) | 78.76 (2.69) | 1.42 | -4.08, 6.93 | 0.610 |
|  | 5M | 80.93 (2.66) | 81.46 (2.72) | -0.94 | -6.49, 4.61 | 0.739 |
| KOOS Symptoms | BL | 66.92 (2.89) | 64.37 (2.92) | - |  |  |
|  | 2M | 73.99 (2.90) | 72.88 (2.94) | -1.43 | -7.38, 4.51 | 0.635 |
|  | 5M | 75.13 (2.90) | 74.52 (2.97) | -1.94 | -7.94, 4.06 | 0.523 |
| KOOS Activities of Daily Living | BL | 73.60 (2.42) | 75.47 (2.45) | - |  |  |
|  | 2M | 82.41 (2.44) | 81.70 (2.47) | 2.58 | -3.06, 8.23 | 0.367 |
|  | 5M | 83.35 (2.44) | 83.48 (2.50) | 1.73 | -3.97, 7.42 | 0.550 |
| KOOS Sport and Recreation | BL | 57.86 (4.50) | 53.78 (4.56) | - |  |  |
|  | 2M | 62.40 (4.55) | 59.43 (4.60) | -1.10 | -14.52, 12.32 | 0.872 |
|  | 5M | 65.82 (4.55) | 68.48 (4.69) | -6.74 | -20.27, 6.80 | 0.327 |
| KOOS QoL | BL | 53.42 (3.43) | 48.02 (3.48) | - |  |  |
|  | 2M | 64.58 (3.46) | 59.10 (3.50) | 0.08 | -7.72, 7.88 | 0.985 |
|  | 5M | 66.56 (3.46) | 63.63 (3.54) | -2.47 | -10.35, 5.40 | 0.536 |
| PROMIS Global Mental, T-score | BL | 52.78 (1.37) | 51.02 (1.39) | - |  |  |
|  | 2M | 53.89 (1.38) | 49.75 (1.40) | 2.38 | -0.09, 4.84 | 0.059 |
|  | 5M | 53.49 (1.38) | 50.45 (1.41) | 1.28 | -1.21, 3.77 | 0.311 |
| PROMIS Global Physical, T-score | BL | 45.95 (1.24) | 45.65 (1.25) | - |  |  |
|  | 2M | 49.05 (1.25) | 48.11 (1.26) | 0.64 | -2.06, 3.34 | 0.641 |
|  | 5M | 49.17 (1.25) | 48.74 (1.28) | 0.13 | -2.59, 2.86 | 0.924 |
| PROMIS Sleep, T-score | BL | 50.05 (1.23) | 54.09 (1.24) | - |  |  |
|  | 2M | 47.09 (1.24) | 49.31 (1.25) | 1.81 | -1.53, 5.14 | 0.286 |
|  | 5M | 47.88 (1.24) | 48.45 (1.27) | 3.46 | 0.10, 6.83 | 0.044 |
| PROMIS Mobility, T-score | BL | 40.84 (0.85) | 40.76 (0.86) | - |  |  |
|  | 2M | 42.49 (0.86) | 43.02 (0.87) | -0.61 | -2.60, 1.38 | 0.548 |
|  | 5M | 43.06 (0.86) | 43.38 (0.88) | -0.40 | -2.41, 1.61 | 0.697 |
| BL: Baseline. 2M: 2 months. 5M: 5 months. KOOS: Knee Injury and Osteoarthritis Outcome Score. Higher KOOS scores indicate more knee issues. PROMIS: Patient-Reported Outcomes Measurement Information System. PROMIS scores above 50 indicate better outcomes. | | | | | | |
